# Intensive Debulking Chemotherapy Improves the Short-Term and Long-Term Efficacy of Anti-CD19-CAR-T in Refractory/Relapsed DLBCL With High Tumor Bulk

**DOI:** 10.3389/fonc.2021.706087

**Published:** 2021-07-30

**Authors:** Cuicui Lyu, Rui Cui, Jia Wang, Nan Mou, Yanyu Jiang, Wei Li, Qi Deng

**Affiliations:** ^1^Department of Hematology, Tianjin First Central Hospital, School of Medicine, Nankai University, Tianjin, China; ^2^Department of Cell Therapy Platform, Shanghai Genbase Biotechnology Co., Ltd, Shanghai, China; ^3^Department of Lymphoma, Key Laboratory of Cancer Prevention and Therapy, Tianjin Medical University Cancer Institute and Hospital, National Clinical Research Center of Cancer, Tianjin, China

**Keywords:** chimeric antigen receptor, diffuse large B-cell lymphoma, refractory, relapse, debulking chemotherapy, high tumor bulk

## Abstract

Anti-CD19 chimeric antigen receptor T (CAR-T) therapy has achieved remarkable effects in refractory/relapsed (R/R) diffuse large B-cell lymphoma (DLBCL). However, when high tumor bulk occurs, patients tend to early progression after CAR-T therapy. Here, we investigated whether pretreatment with intensive debulking chemotherapy could improve the outcome of CAR-T in such patients. Fifty-seven patients with R/R DLBCL were enrolled, and 42 patients received anti-CD19-CAR-T therapy, among which, 25 patients (the combined group) with high tumor bulk received debulking chemotherapy and anti-CD19-CAR-T therapy sequentially. Another 17 patients (the control group) without high tumor bulk received anti-CD19-CAR-T therapy only. According to the response to debulking chemotherapy, patients of the combined group were divided into chemo-sensitive and chemo-refractory groups. Within 2 months, the objective response rate (ORR) was higher in the chemo-sensitive group than in the chemo-refractory group (*P* = 0.031). Grades 1–3 cytokine release syndrome (CRS) was reported, and no difference was shown in CRS grade distribution between the chemo-sensitive and chemo-refractory groups (*P* = 0.514). The chemo-sensitive group demonstrated longer overall survival (OS) than the chemo-refractory group (*P* = 0.042). Of the chemo-sensitive group, the 1-year disease free survival (DFS) and OS rates were 52.6 and 57.9%, respectively. Besides, no significant differences were found in ORR, DFS, and OS between the chemo-sensitive and control groups (ORR: *P* = 0.593; DFS: *P* = 0.762; OS: *P* = 0.531). In summary, effective debulking chemotherapy improved the short-term ORR and long-term OS of CAR-T therapy in R/R DLBCL with high tumor bulk, with outcomes comparable to those of R/R DLBCL without high tumor bulk. The clinical trial of our study was registered at http://www.chictr.org.cn/index.aspx as *ChiCTR-ONN-16009862* and *ChiCTR1800019622*.

**Clinical Trial Registration:**

http://www.chictr.org.cn/index.aspx, identifier (*ChiCTR-ONN-16009862* and *ChiCTR1800019622)*.

## Introduction

Refractory/relapsed (R/R) diffuse large B-cell lymphoma (DLBCL) has poor therapeutic response and prognosis. The standard treatment for R/R DLBCL involves high-dose chemotherapy followed by autologous stem cell transplantation (1). However, the long-term remission rate associated with such treatment is only 10–20% (2, 3).

Anti-CD19 chimeric antigen receptor T (CAR-T) therapy has shown significant effects in B-cell malignancies. Data from the ZUMA-1 and JULIET studies have reported objective response rates (ORR) of 50–80% and complete response (CR) rates of 30–50% in B-cell lymphomas (4, 5). The NCCN guidelines (version 4.2019) have recommended CAR-T therapy for DLBCL patients achieving partial response (PR) following second-line therapy and for those with disease relapse after achieving CR to second-line therapy or progressive disease (PD).

CAR-T therapy is often associated with potentially fatal toxicities, including cytokine release syndrome (CRS) and neurologic toxicities, especially in cases with high tumor burden (6–8). In addition, high metabolic tumor volume has been identified as a predictive factor of early progression in CAR-T therapy for DLBCL (9).

Radiotherapy has been used to debulk tumor burden before the initiation of anti-CD19-CAR-T therapy and has shown some efficacy (10, 11). For patients with lymphoma who are not suitable for local radiotherapy, we hypothesized that the efficacy of anti-CD19-CAR-T therapy may be improved by reducing the tumor burden with intensive chemotherapy beforehand. We had previously proved that anti-CD19-CAR-T therapy in R/R B-cell acute lymphoblastic leukemia (B-ALL) was associated with high treatment response and manageable toxicities following intensive lymphodepleting chemotherapy (12).

Intensive chemotherapy is often used for R/R DLBCL with regimens, such as DHAP (dexamethasone, cisplatin, and cytarabine) (13), DA-EPOCH (dose-adjusted etoposide, prednisone, vincristine, cyclophosphamide, and doxorubicin) (14, 15), ICE (ifosfamide, carboplatin, and etoposide) (16–18) and GemOx (gemcitabine and oxaliplatin) (19–22). Since many patients with R/R DLBCL with high tumor bulk have experienced a transient reduction in tumor burden after intensive chemotherapy, we hypothesized that early treatment to reduce the tumor burden in such patients would improve the curative effects of anti-CD19-CAR-T therapy.

Our study therefore aimed to investigate the effects of intensive debulking therapy on the outcomes of anti-CD19-CAR-T therapy in patients with R/R DLBCL with high tumor bulk. In our study, twenty-five such patients were enrolled and underwent humanized anti-CD19-CAR-T therapy following intensive chemotherapy. Our results demonstrated that effective debulking chemotherapy improved the short-term ORR and long-term overall survival (OS) of anti-CD19-CAR-T therapy in such patients.

## Patients and Methods

### Study Participants

Our study was a single-center retrospective study involving patients with R/R DLBCL. The R/R DLBCL diagnosis was made based on the 2008 World Health Organization guidelines. To be eligible for enrollment, patients had to previously receive at least two lines of therapy and either had a relapse after or were ineligible for autologous transplantation. Patients were enrolled in clinical trials of anti-CD19-CAR-T cells expressing humanized anti-CD19 scFv and 4-1BB-CD3ζ costimulatory-activation domains (ChiCTR-ONN-16009862 and ChiCTR1800019622). Patients were excluded if they had previously received anti-CD19-CAR-T therapy. The study was approved by the Ethics Committee of Tianjin First Central Hospital, and all participants provided written informed consent in accordance with the Declaration of Helsinki. Follow-ups were performed from the date of anti-CD19-CAR-T cell infusion to either the cutoff date (November 30, 2020) or the date of death.

### Generation and Detection of Anti-CD19-CAR-T Cells

CAR-T cells were manufactured as described in our previous study (23). The detailed methods were as follows: peripheral blood mononuclear cells (PBMCs) were collected and isolated by Ficoll density gradient centrifugation. CD3^+^ T cells were selected by CD3 microbeads (Miltenyi Biotec, Cambridge, MA, USA), stimulated by anti-CD3/anti-CD28 mAb-coated Human T-Expander beads (Cat.no. 11141D; Thermo Fisher Scientific, Waltham, MA, USA) and cultured in T-cell medium *X-Vivo* 15 (Lonza Group, Ltd., Basel, Switzerland) supplemented with 250 IU/ml interleukin-2 (IL-2; Proleukin; Novartis International AG, Basel, Switzerland). All the CD3^+^ T cells (3 × 10^6^) were transduced with a lentiviral vector encoding humanized CD19 CAR constructs (10 μg, lenti-CD19-2rd-CAR; Shanghai Genbase Biotechnology Co., Ltd., Shanghai, China) and cultured in media containing recombinant human IL-2 (250 IU/ml). On the 12th day of cultivation, transduction efficiencies of anti-CD19-CAR were analyzed by flow cytometry (FCM) (BD Biosciences, San Jose, CA, USA).

### Debulking Chemotherapy and Anti-CD19-CAR-T Cell Infusion

**Figure 1:**
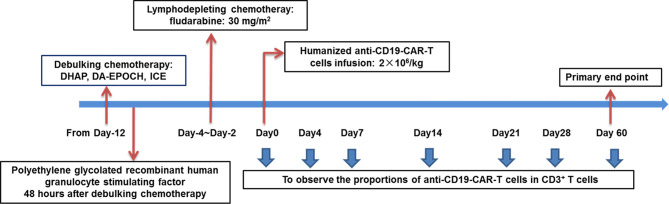
Schematic diagram showing the strategy for the treatment of patients in the combined group. Patients were given debulking chemotherapy and lymphodepleting chemotherapy sequentially, and then received anti-CD19-CAR-T therapy.

To decrease tumor burden, patients with high tumor bulk were first subjected to intensive debulking chemotherapy after leukapheresis. The chemotherapy regimens contained DHAP, DA-EPOCH, and ICE, to which the patients were previously sensitive. Polyethylene-glycolated recombinant human colony granulocyte stimulating factor therapy (6 mg) was administered to all patients 48 h after debulking chemotherapy. Patients were given recombinant human thrombopoietin or recombinant human interkeulin-11 to prevent the occurrence of severe thromocytopenia. From day −4 to day −2, patients with high tumor bulk received fludarabine (30 mg/m^2^), whereas those without high tumor bulk (the control group) received fludarabine (30 mg/m^2^) and cyclophosphamide (400 mg/m^2^) as lymphodepleting chemotherapy. Humanized anti-CD19-CAR-T cell infusion (2 × 10^6^/kg) was eventually initiated on day 0 (The day on which CAR-T cells were infused was numbered as day 0.) (**Figure 1**).

### The Efficacies of Intensive Debulking Chemotherapy and Subsequent Anti-CD19-CAR-T Therapy

The efficacy of intensive debulking chemotherapy was evaluated on the day of anti-CD19-CAR-T cell infusion (before infusion) *via* computed tomography examination, whereas that of anti-CD19-CAR-T therapy was evaluated within 2 months of initiation. The curative effects of both therapies were assessed according to the Lugano Revised Criteria for Response Assessment (24). This clinical response standard included CR, PR, stable disease (SD), and PD. The primary end point was the ORR within 2 months after anti-CD19-CAR-T cell infusion. Secondary end points included adverse events, disease free survival (DFS), and OS.

### Adverse Events

The grade of CRS was determined as previously described (25). Adverse events were graded according to the Common Terminology Criteria for Adverse Events, version 4.03, set by the US Department of Health and Human Services. Immune effector cell-associated neurotoxic syndrome (ICANS) grades were evaluated in accordance with the American Society for Blood and Marrow Transplantation (ASBM) ICANS Consensus Grading for Adults (26).

Infectious complications were evaluated on the basis of body temperature and infectious biomarkers such as high-sensitivity C-reactive protein (hs-CRP), procalcitonin (PCT), 1,3-beta-D glucan (G test), and Galactomannan antigen (GM test). Epstein–Barr virus testing and cytomegalovirus DNA testing were also performed in some patients.

### Serum Cytokine Levels After Anti-CD19-CAR-T Cell Infusion

Serum cytokine levels were measured on days 0, 4, 7, 14, and 21 using the double antibody one-step sandwich enzyme-linked immunosorbent assay (ELISA) method. The measured cytokines included interleukin-2R (IL-2R), IL-6, IL-10, and tumor necrosis factor-α (TNF-α).

### Expansion and Persistence of Anti-CD19-CAR-T Cells

The proportion of anti-CD19-CAR-T cells in CD3^+^ T cells was observed by FCM on days 0, 4, 7, 14, 21, 28, and 60 after anti-CD19-CAR-T cell infusion.

### Statistical Analysis

Statistical analyses were performed with GraphPad Prism 5 (GraphPad Software, San Diego, CA, USA). All values were expressed as mean ± SD unless otherwise indicated. Unpaired Student’s t-test was used to compare quantitative values between groups. Categorical variables were compared by χ^2^ test or Fisher’s exact test. CRS was compared by Mann–Whitney rank. The probabilities of DFS and OS were estimated using the Kaplan–Meier method and were compared using the log-rank test. *P <*0.05 was considered statistically significant.

## Results

### Patient Characteristics

**Figure 2:**
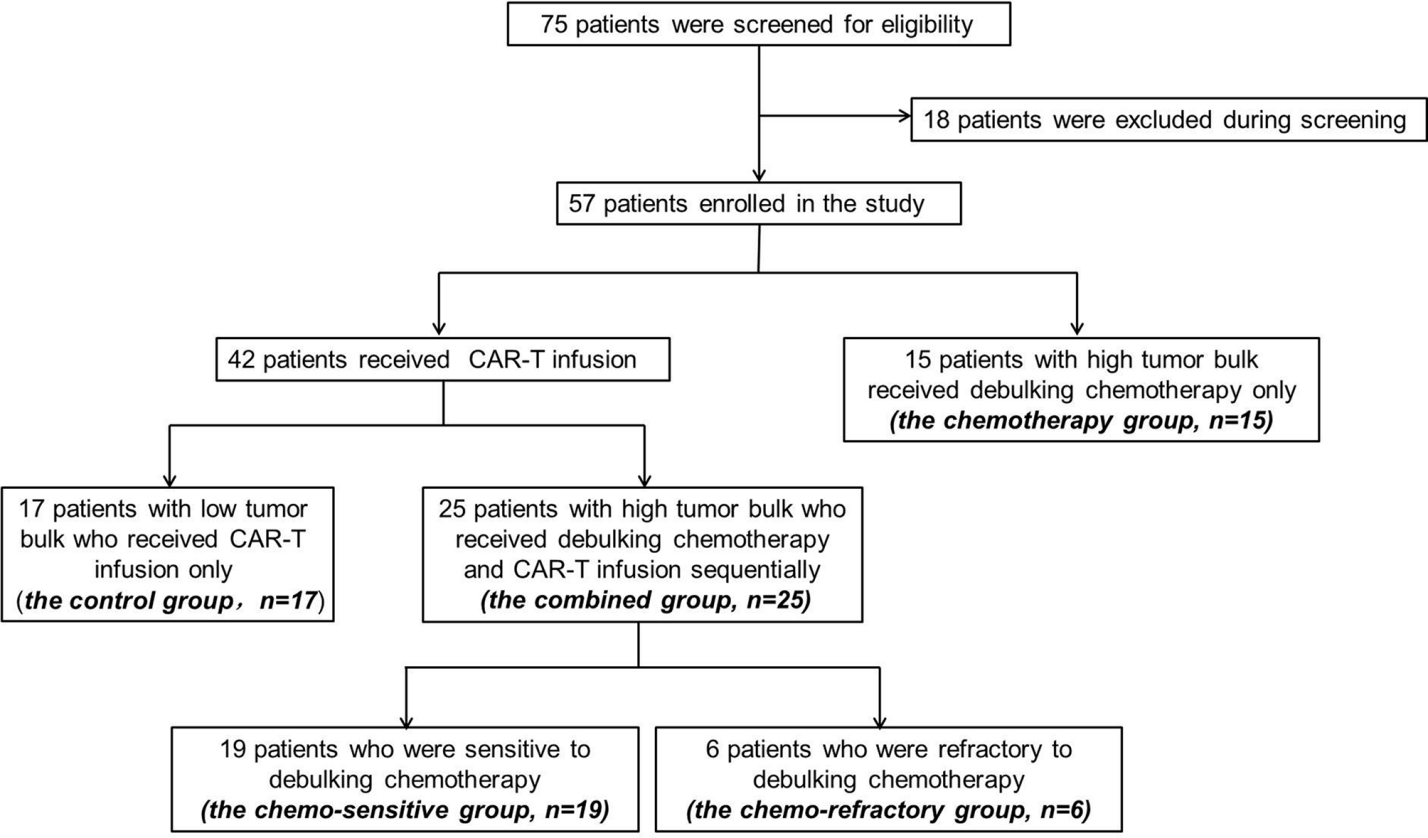
Screening, enrollment, and treatment of patients. Among the 75 patients under screening, 18 decided not to participate. Among the 57 enrolled patients, 15 patients with high tumor bulk received debulking chemotherapy only and declined further CAR-T participation.

Between September 2018 and August 2020, 75 patients were screened, and 57 were enrolled. Of the enrolled patients, 15 patients (the chemotherapy group) with high tumor bulk did not participate in anti-CD19-CAR-T therapy after debulking chemotherapy due to personal preference. The remaining 42 (79%) patients received anti-CD19-CAR-T therapy, among which, 25 patients (the combined group) had at least one report of high tumor bulk (tumor diameter ≥7.5 cm), another 17 patients (the control group) without high tumor bulk who received the same anti-CD19-CAR-T therapy were included as controls (**Figure 2**). The baseline characteristics of patients in the combined group are presented in **Table 1**. Other than the maximum diameter, there were no differences in age, sex, cell origin of cancer, stage, IPI scores, primary lines of chemotherapy between the combined and control groups (**Table 2**). No significant differences of baseline characteristics were found between the chemotherapy and combined groups (**Supplemental Table S1**).

**Table 1:** Baseline characteristics of patients in the combined group.

Patient number	Age	Sex	Molecular subtype	Stage	Previous response status	IPI at enrollment	Previous lines of therapy	Maximum tumor diameter (cm)
P1^#^	31	male	Non-GCB	III	refractory	3	3	7.9
P2^#^	56	male	GCB	IV	relapse	3	2	7.8
P3^#^	15	male	GCB	IV	relapse	4	4	8.6
P4^#^	54	male	GCB	III	refractory	3	2	8.5
P5^#^	35	male	Non-GCB	III	relapse	3	3	7.9
P6^#^	52	male	Non-GCB	IV	relapse	3	4	9.4
P7^#^	61	male	GCB	III	refractory	3	3	8.3
P8^#^	51	male	GCB	III	refractory	3	2	8.0
P9^#^	62	male	GCB	IV	refractory	4	3	11.5
P10^#^	52	female	Non-GCB	IV	relapse	3	2	7.5
P11^#^	70	male	GCB	III	relapse	2	4	7.7
P12^#^	41	male	GCB	IV	relapse	3	3	8.3
P13^#^	50	male	Non-GCB	IV	relapse	2	4	8.6
P14^#^	39	female	Non-GCB	IV	refractory	3	2	13.4
P15^#^	63	male	Non-GCB	IV	refractory	5	3	10.0
P16^#^	50	female	Non-GCB	IV	refractory	3	4	11.5
P17^#^	60	female	GCB	IV	refractory	3	4	8.3
P18^#^	68	male	Non-GCB	IV	refractory	4	2	13.0
P19^#^	44	male	Non-GCB	IV	relapse	3	3	9.0
P20^#^	77	male	Non-GCB	III	refractory	4	2	15.0
P21^#^	46	female	GCB	IV	refractory	2	4	14.0
P22^#^	33	male	Non-GCB	IV	refractory	2	3	12.0
P23^#^	41	female	GCB	IV	refractory	3	3	8.8
P24^#^	46	female	Non-GCB	IV	refractory	3	3	20.0
P25^#^	77	male	Non-GCB	IV	relapse	3	2	11.7

**Table 2:** Comparison of baseline characteristics of patients between the combined and control groups.

Characteristics	The combined group	The control group	*P*-value
***Median age (range)—year***	51 (15–77)	66 (19–76)	0.0934
***Male—no. (%)***	18 (72.0)	9 (52.9)	0.326
***Disease stage at study entry—no. (%)***			0.059
**Stage I/II**	0 (0)	3 (17.6)	
**Stage III/IV**	25 (100.0)	14 (82.4)	
***Cell origin of cancer—no. (%)***			0.222
**Germinal center B-cell type**	11 (44.0)	11 (64.7)	
**Non-germinal center B-cell type**	14 (56.0)	6 (35.3)	
***Relapse after last therapy—no. (%)***	10 (40.0)	10 (58.8)	1.000
***Refractory DLBCL—no. (%)***	15 (60.0)	7 (41.2)	1.000
***No. of previous lines of antineoplastic therapy—no. (%)***			0.353
**1–2**	8 (32.0)	8 (47.1%)	
**3–4**	17 (68.0)	9 (52.9%)	
***IPI scores at study entry—no. (%)***			0.268
**0–2 points**	4 (16.0)	6 (47.1%)	
**3–5 points**	21 (84.0)	11 (52.9%)	
***Medium tumor diameter (range)—cm***	10.0 (7.5–20.0)	4.1 (2.7–7.0)	<0.0001

### Transduction Efficiency, Amplification, and Infusion of Anti-CD19-CAR-T Cells

The mean anti-CD19-CAR transduction efficiencies in the final products were 55.21 ± 12.03% and 57.45 ± 11.35% in the combined and control groups, respectively (*P* = 0.3752). After 12–15 days of culture, the mean number of anti-CD19-CAR-T cells was (8.30 ± 3.42) × 10^6^ cells/kg and (7.69 ± 3.28) ×10^6^ cells/kg in the two groups, respectively (*P* = 0.4387). The mean dose of humanized anti-CD19-CAR-T cell infusion on day 0 was (2.06 ± 0.44) ×10^6^ cells/kg.

### Efficacy and Toxicities of Intensive Debulking Chemotherapy

In the combined group, significant shrinkage in tumor bulk with concomitant relief in clinical symptoms was observed in 19 (19/25) patients (the chemo-sensitive group), although PR was not reached [≥50% decrease in the sum of the products of the greatest diameters (SPD) in less than six target measurable nodes and extranodal sites]. In contrast, tumor bulk shrinkage was not observed in the other six (6/25) patients (the chemo-refractory group), with the stage of SD (**Supplemental Table S2**). Details of the intensive debulking chemotherapy regimen are shown in **Supplemental Table S2**. There were no significant differences in age, sex, cell origin of cancer, stage, IPI scores, primary lines of chemotherapy, and maximum tumor diameter between the chemo-sensitive and chemo-refractory groups (**Supplemental Table S3**).

In the chemotherapy group, the intensive debulking chemotherapy regimen included DHAP (8/15), DA-EPOCH (4/15), and ICE (3/15). Significant shrinkage in tumor bulk with concomitant relief in clinical symptoms was observed in 11 (11/15) patients, whereas tumor bulk shrinkage was not observed in other 4 (4/15) patients. There was no difference in the percentage of sensitivity to chemotherapy between the chemotherapy and combined groups (*P* = 1.000).

Debulking chemotherapy was well-tolerated in all patients. In the combined group, only one patient (P19^#^) reported hypocalcemia, hyperphosphatemia, and high levels of blood urea nitrogen, creatinine, and uric acid after debulking chemotherapy, which all returned to normal soon. No tumor lysis syndrome occurred in the remaining 24 patients, and their maximum serum potassium, calcium, phosphorus, blood urea nitrogen, creatinine, and uric acid levels remained within normal ranges (**Supplemental Table S2**). Besides, five patients had grade 3/4 neutropenia, four had grade 3/4 thrombocytopenia, and three had grade 3/4 anemia (**Supplemental Table S4**). Only two patients with grade 3/4 neutropenia were diagnosed with gram-negative bacterial infections, which were cured by anti-infective therapy.

In the chemotherapy group, no tumor lysis syndrome occurred, and four patients had grade 3/4 neutropenia, four had grade 3/4 thrombocytopenia, and two had grade 3/4 anemia. Three patients with grade 3/4 neutropenia were diagnosed with gram-negative bacterial infections, which were cured by anti-infective therapy.

### Clinical Response of Anti-CD19-CAR-T Therapy

**Figure 3:**
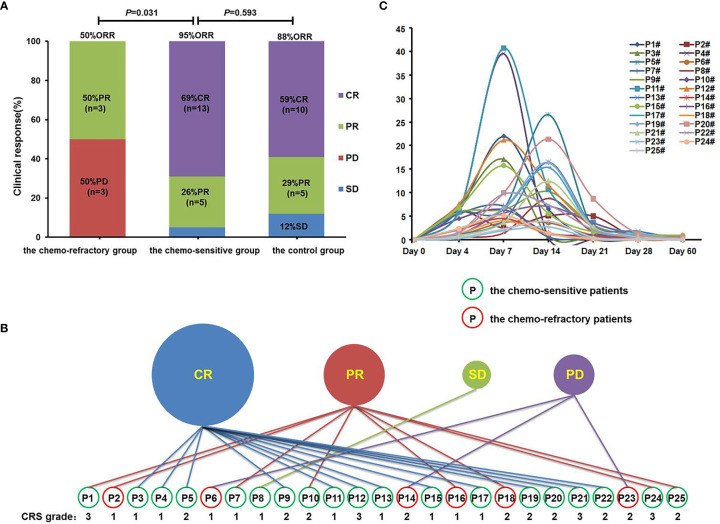
Clinical response, CRS grades, and proportions of anti-CD19-CAR-T cells. **(A)** Clinical responses were shown in histogram. ORR, objective response rate; CR, complete response; PR, partial response; SD, stable disease; PD, progressive disease. **(B)** Clinical response and CRS grades of patients in the combined group. Patients in the green circle were sensitive to the debulking chemotherapy, whereas patients in the red circle did not. **(C)** The proportions of anti-CD19-CAR-T cells of patients in the combined group on days 0, 4, 7, 14, 21, 28, and 60.

All patients were evaluated within 2 months of anti-CD19-CAR-T cell infusion. The chemo-sensitive group achieved an ORR of 95.0% (18/19) and a CR rate of 68.4% (13/19). The chemo-refractory group reported an ORR of 50.0% (3/6), with three patients achieving PR and three achieving PD (**Figure 3A**). The ORR was significantly higher in the chemo-sensitive group than in the chemo-refractory group (*P* = 0.031). In the control group, the ORR was 88% (15/17), which was not significantly different from that of the chemo-sensitive group (*P* = 0.593) (**Figure 3A**).

It was worth noting that among the 25 patients in the combined group, all patients with CR responded to debulking chemotherapy, whereas all patients with PD did not (*P* = 0.002). This suggests that only patients who have achieved CR benefited from debulking chemotherapy, while the PD patients did not.

### Adverse Events of Anti-CD19-CAR-T Therapy

**Figure 4:**
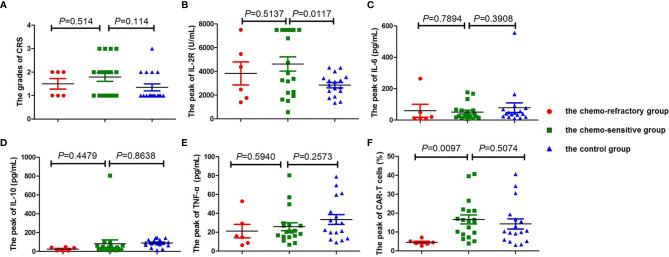
Comparison of CRS, cytokine levels, and the peak of CAR-T cells between the chemo-sensitive, chemo-refractory, and control groups. No significant differences were found in CRS (**A**, *P* = 0.514), IL-2R (**B**, *P* = 0.5137), IL-6 (**C**, *P* = 0.7894), IL-10 (**D**, *P* = 0.4479), and TNF-α (**E**, *P*=0.5940) between the chemo-sensitive and chemo-refractory groups, whereas statistical differences were observed in the peak of CAR-T cells between the two groups (**F**, *P* = 0.0417). No significant differences were found in CRS (**A**, *P* = 0.114), IL-6 (**C**, *P* = 0.3908), IL-10 (**D**, *P* = 0.8638), TNF-α (**E**, *P* = 0.2573), and the peak of CAR-T cells (**F**, *P* = 0.5074) between the chemo-sensitive and control groups, whereas statistical differences were observed in IL-2R between the two groups (**B**, *P* = 0.0117). CRS was compared by Mann–Whitney rank. Unpaired Student’s t-test was used to compare the cytokine levels and the peak of CAR-T cells.

Among the patients in the combined group, four who achieved CR or PR following anti-CD19-CAR-T therapy were diagnosed as having grade 3 CRS, whereas all other patients developed grade 1–2 CRS (**Figure 3B**). No patients developed ICANS, and no patients died of CRS or ICANS. There were no significant differences in the distribution of CRS grades between the chemo-sensitive and chemo-refractory groups (*P* = 0.514) (**Figure 4A**). In the control group, twelve patients had grade 1 CRS, four had grade 2 CRS, and one had grade 3 CRS. Similarly, there were no significant differences in the CRS grade distribution between the chemo-sensitive and control groups (*P* = 0.114) (**Figure 4A**).

During anti-CD19-CAR-T therapy, symptoms, such as fever, tachycardia, tachypnea, nausea, vomiting, and diarrhea were reported. Cases of hepatotoxicity and kidney injury were mild and transient. Among the patients in the combined group, twenty-one patients had grade 3/4 neutropenia, eleven had grade 3/4 thrombocytopenia, and eight had grade 3/4 anemia (**Table 3** and **Supplemental Table S4**). Hematological toxicities were recovered within 2 weeks. There were no significant differences in the incidences of grade 3/4 hematological toxicities between the chemo-sensitive and chemo-refractory groups (neutropenia: *P* = 0.694; thrombocytopenia: *P* = 0.548; anemia: *P* = 0.651). In the chemo-sensitive group, significantly higher incidence of grade 3/4 neutropenia was observed among patients with CR than those with PD and SD (*P* = 0.007).

**Table 3:** Notable adverse events of anti-CD19-CAR-T therapy in patients of the combined group.

Events	
***Vital signs***	
**Temperature ≥38°C (fever)**	21 (84%)
**Systolic blood pressure <90 mm Hg (hypotension)**	0 (0%)
**Needing oxygen for SaO2 >90% (hypoxia)**	0 (0%)
***Organ toxicities***	
***Cardiac***	
**Tachycardia**	18 (72%)
**Arrhythmias**	0 (0%)
**Heart block**	0 (0%)
**Low ejection fraction**	0 (0%)
***Respiratory***	
**Tachypnea**	21 (84%)
**Pleural effusion**	0 (0%)
**Pulmonary edema**	0 (0%)
***Gastrointestinal***	
**Nausea**	5 (20%)
**Vomiting**	3 (12%)
**Diarrhea**	1 (4%)
***Hepatic***	
**Increased serum ALT, AST**	8 (32%)
** Increased serum bilirubin levels**	3 (12%)
***Renal***	
** Acute kidney injury (increased serum creatinine levels)**	5 (20%)
**Decreased urine output**	2 (8%)
***Dermatological***	
**Rash**	2 (8%)
***Coagulopathy***	
**Disseminated intravascular coagulation**	0 (0%)
***Neurological***	0 (0%)
***Hematological***	
**Neutropenia (grade 3/4)**	21 (84%)
** Thrombocytopenia (grade 3/4)**	11 (44%)
**Anemia (grade 3/4)**	8 (32%)

Among the patients in the combined group, 84% (21/25) had fever (temperature ≥ 38°C) following anti-CD19-CAR-T cell infusion. One patient (P8^#^) was diagnosed with *Staphylococcus epidermidis* infection and was successfully treated with anti-infection treatment. Three patients (P9^#^, P10^#^, and P14^#^) developed fever with elevated hs-CRP and PCT levels, but without specific pathogens identified. The remaining 17 patients developed fever without elevated PCT levels, with negative G and GM test results, and with no evidence of infection. Invasive fungal diseases were not observed in any of the patients.

### Serum Cytokine Levels Following Anti-CD19-CAR-T Cell Infusion

The serum levels of IL-2R, IL-6, IL-10, and TNF-α peaked from days 4 to 7, and subsequently declined from days 12 to 14 after the anti-CD19-CAR-T cell infusion. There were no significant differences between the chemo-sensitive and chemo-refractory groups (IL-2R: *P* = 0.5137; IL-6: *P* = 0.7894; IL-10: *P* = 0.4479; TNF-α: *P* = 0.5940, **Figures 4B–E**). Serum IL-2R levels were significantly higher in the chemo-sensitive group than in the control group (*P* = 0.0117), whereas no differences were found in serum IL-6, IL-10, and TNF-α levels between these two groups (IL-6: *P* = 0.3908; IL-10: *P* = 0.8638; TNF-α: *P* = 0.2573, **Figures 4B–E**).

### Expansion of Anti-CD19-CAR-T Cells

The proportion of anti-CD19-CAR-T cells in CD3^+^ T cells was observed throughout the anti-CD19-CAR-T therapy. Among the patients in the combined group, the average peak of anti-CD19-CAR-T cells was 13.74 ± 10.45% on days 7–14. This dropped to 0.64 ± 0.51% on day 28, and further declined to 0.20 ± 0.26% on day 60 (**Figure 3C**). The average peak of anti-CD19-CAR-T cells was higher in the chemo-sensitive group than in the chemo-refractory group (16.65 *vs* 4.51%, *P* = 0.0097), whereas no differences were observed between the chemo-sensitive and control groups (*P* = 0.5074) (**Figure 4F**).

### Follow-Up

**Figure 5:**
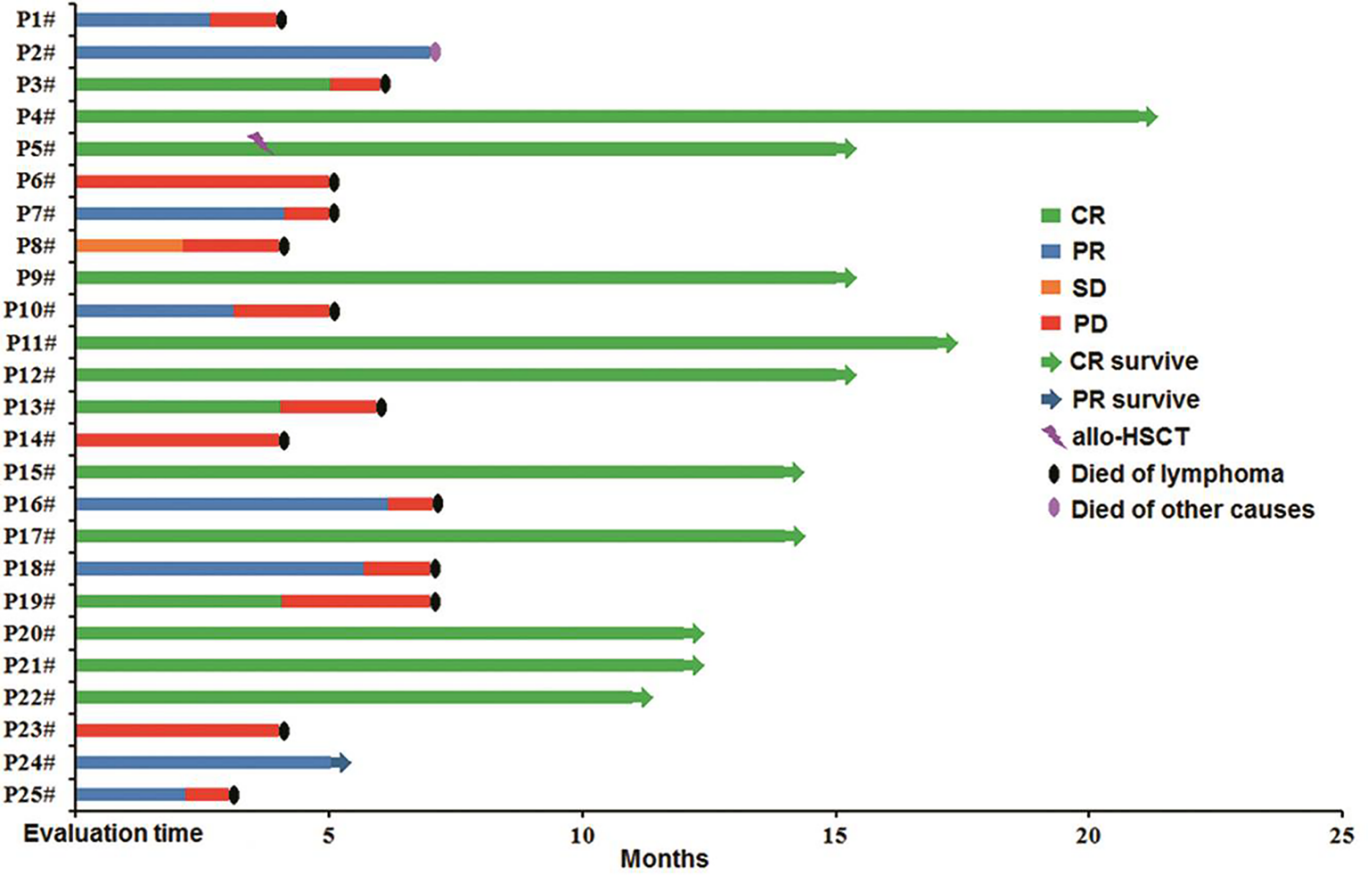
Follow-up of patients in the combined group. P4^#^, P5^#^, P9^#^, P11^#^, P12^#^, P15^#^, P17^#^, P20^#^, P21^#^, and P22^#^ maintained CR, and allo-HSCT was carried out in P5^#^; P24^#^ maintained PR; P1^#^, P3^#^, P6^#^, P7^#^, P8^#^, P10^#^, P13^#^, P14^#^, P16^#^, P18^#^, P19^#^, P23^#^, and P25^#^ died of the original disease, P2^#^ died of other factors. HSCT, hematopoietic stem cell transplantation.

The median follow-up time was 7 months (range: 3–21 months). Among the patients in the combined group who achieved CR during anti-CD19-CAR-T therapy, ten patients (10/13) survived until the cutoff date, whereas three (3/13) died of disease relapse. Only one (1/8) patient who achieved PR following this combination therapy survived until the cutoff date. All patients who achieved SD and PD died of disease progression (**Figure 5**).

**Figure 6:**
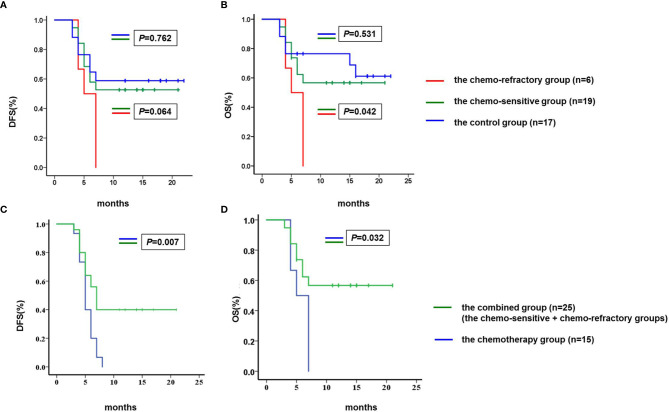
Comparison of DFS and OS. **(A)** The median DFS of the chemo-sensitive and control groups was not achieved, and the median DFS of the chemo-refractory group was 5.0 months (95% confidence interval 3.2–6.8). No differences were observed in DFS between the chemo-sensitive and chemo-refractory groups (*P* = 0.064), neither between the chemo-sensitive and control groups (*P* = 0.762). **(B)** The median OS of the chemo-sensitive and control groups was not achieved, and the median OS of the chemo-refractory group was 5.0 months (95% confidence interval 3.2–6.8). The median OS of the chemo-sensitive group was longer than that of the chemo-refractory group (*P* = 0.042), no differences were observed in OS between the chemo-sensitive and control groups (*P* = 0.531). **(C)** The median DFS of the chemotherapy and combined groups was 5 months (95% confidence interval 4.3–5.7) and 7 months (95% confidence interval 5.8–8.2), respectively (*P* = 0.007). **(D)** The median OS of the chemotherapy and combined groups was 5 months (95% confidence interval 4.3–5.7) and 7 months (95% confidence interval 5.8–8.2), respectively (*P* = 0.032). DFS and OS were estimated using the Kaplan–Meier method and were compared using the log-rank test. DFS, disease free survival; OS, overall survival.

By the cutoff date, both median DFS and OS were not achieved in the chemo-sensitive and control groups. Of the chemo-sensitive group, the 1-year DFS and OS rates were 52.6 and 57.9%, respectively. The median OS of the chemo-sensitive group was longer than that of the chemo-refractory group (*P* = 0.042), whereas there were no differences in DFS between the two groups (*P* = 0.064). Furthermore, no significant differences in DFS and OS were observed between the chemo-sensitive and control groups (DFS: *P* = 0.762; OS: *P* = 0.531) (**Figures 6A, B**). Besides, DFS and OS of the combined group were significantly longer than that of the chemotherapy group (DFS: *P* = 0.007; OS: *P* = 0.032) (**Figures 6C, D**).

## Discussion

CAR-T therapy represents a novel treatment choice for patients with R/R DLBCL. As previous correlative analyses have largely focused on the toxicity of CAR-T therapy, the factors associated with the durable response of such treatment remain incompletely elucidated. The number of treatment lines before CAR-T therapy and C-reactive protein (CRP) >30 mg/l at the time of lymphodepletion has been reported to associate with poor progression-free survival (PFS) in R/R aggressive B-cell lymphoma, and CRP >30 mg/L further associated with shorter OS (27). Patients with poor Eastern Cooperative Oncology Group performance status scores of 2–4 and elevated lactate dehydrogenase levels had shorter PFS and OS (28). The ZUMA-1 study demonstrated that durable treatment response was associated with low baseline tumor burden, low systemic inflammation, and high CCR7^+^CD45RA^+^ T cell product in patients with refractory large B-cell lymphoma treated with axicabtagene ciloleucel (29). Another study demonstrated that patients with R/R DLBCL with high tumor burden experienced poor long-term outcomes after CAR-T therapy and tended to develop early progression (9).

In our clinical study of patients with R/R DLBCL with high tumor bulk, intensive chemotherapy was administered to debulk tumor burden prior to the anti-CD19-CAR-T therapy. We found that the chemo-sensitive group with decreased tumor burden demonstrated higher ORR within 2 months and longer OS than the chemo-refractory group. Besides, no differences were found in ORR, DFS, and OS between the chemo-sensitive and control groups, indicating that the short- and long-term efficacy of anti-CD19-CAR-T therapy of the chemo-sensitive group was comparable to those of the control group. This suggests that effective debulking chemotherapy improves the short- and long-term efficacy of anti-CD19-CAR-T therapy in R/R DLBCL with high tumor bulk.

Besides, in our study, 15 patients (the chemotherapy group) with high tumor bulk received debulking chemotherapy only, and 25 patients with high tumor bulk received debulking chemotherapy and CAR-T infusion sequentially (the combined group). DFS and OS of the combined group were significantly longer than that of the chemotherapy group. This suggests that sole intensive debulking chemotherapy cannot change the outcome of patients with high tumor bulk with R/R DLBCL, whereas intensive debulking chemotherapy combined with CAR-T therapy supplies a new effective treatment regimen for such patients.

Higher tumor burden in the bone marrow has been established as a risk factor for toxicity in both patients with B-ALL receiving anti-CD19-CAR-T therapy (8) and patients with multiple myeloma receiving anti-B-cell maturation antigen (BCMA)-CAR-T therapy (30, 31). No reports currently exist on the association between higher tumor burden and the development of severe CRS and severe neurotoxicities in non-Hodgkin lymphoma (7). In our study, no differences were found in the distribution of CRS grades between the chemo-sensitive and chemo-refractory groups, neither were there any differences in the serum levels of IL-2R, IL-6, IL-10, and TNF-α.

There are currently no reports on the association between severe cytopenia and CAR-T therapy response. In the chemo-sensitive group of our study, the patients with CR had significantly higher percentages of grade 3/4 neutropenia than those with PD and SD. However, these data may be biased by the small sample size, and a larger cohort is hence needed to validate this relationship. Moreover, grade 3/4 neutropenia occurred following debulking chemotherapy in four patients of the chemo-sensitive group and did not return to normal on the day of CAR-T cell infusion, which may represent another bias factor.

In conclusion, our study demonstrated that effective debulking chemotherapy improved the short-term ORR and long-term OS of anti-CD19-CAR-T therapy in patients with R/R DLBCL with high tumor bulk, with outcomes comparable to those of patients with R/R DLBCL without high tumor bulk. Effective debulking chemotherapy should therefore be administered to patients with R/R DLBCL with high tumor bulk prior to anti-CD19-CAR-T therapy to decrease tumor burden and thereby improve the short- and long-term efficacy of anti-CD19-CAR-T therapy. Our study provides an effective treatment strategy for R/R patients with DLBCL with high tumor bulk.

## Data Availability Statement

The original contributions presented in the study are included in the article/**Supplementary Material**. Further inquiries can be directed to the corresponding authors.

## Ethics Statement

The studies involving human participants were reviewed and approved by the Medical Ethics Committee of the Department of Hematology, Tianjin First Center Hospital (Tianjin, China). (Approved No. of ethic committee: 2015002X and 2018N105KY). Written informed consent to participate in this study was provided by the participants’ legal guardian/next of kin.

## Author Contributions

CL analyzed and interpreted data and wrote the manuscript. RC and JW analyzed and interpreted data. NM provided the humanized lenti-CD19-2rd-CAR, and YJ provided feedback on the approach and analyses. QD and WL designed the study, acquired, analyzed, and interpreted data, and wrote the manuscript. All authors contributed to the article and approved the submitted version.

## Funding

The National Natural Science Foundation of China (81900186).

## Conflict of Interest

NM was employed by the company Shanghai Genbase Biotechnology Co., Ltd.

The remaining authors declare that the research was conducted in the absence of any commercial or financial relationships that could be construed as a potential conflict of interest.

## Publisher’s Note

All claims expressed in this article are solely those of the authors and do not necessarily represent those of their affiliated organizations, or those of the publisher, the editors and the reviewers. Any product that may be evaluated in this article, or claim that may be made by its manufacturer, is not guaranteed or endorsed by the publisher.

## References

[1] Chaganti S Illidge T Barrington S McKay P Linton K Cwynarski K. Guidelines for the Management of Diffuse Large B-Cell Lymphoma. Br J Haematol (2016) 174:43–56. 10.1111/bjh.14136 27196701

[2] Crump M Neelapu SS Farooq U Van Den Neste E Kuruvilla J Westin J. Outcomes in Refractory Diffuse Large B-Cell Lymphoma: Results From the International SCHOLAR-1 Study. Blood (2017) 130:1800–8. 10.1182/blood-2017-03-769620 PMC5649550 28774879

[3] Gisselbrecht C Glass B Mounier N Singh Gill D Linch DC Trneny M. Salvage Regimens With Autologous Transplantation for Relapsed Large B-Cell Lymphoma in the Rituximab Era. J Clin Oncol (2010) 28:4184–90. 10.1200/JCO.2010.28.1618 PMC3664033 20660832

[4] Neelapu SS Locke FL Bartlett NL Lekakis LJ Miklos DB Jacobson CA. Axicabtagene Ciloleucel CAR T-Cell Therapy in Refractory Large B-Cell Lymphoma. N Engl J Med (2017) 377:2531–44. 10.1056/NEJMoa1707447 PMC5882485 29226797

[5] Schuster SJ Bishop MR Tam CS Waller EK Borchmann P McGuirk JP. Tisagenlecleucel in Adult Relapsed or Refractory Diffuse Large B-Cell Lymphoma. N Engl J Med (2019) 380:45–56. 10.1056/NEJMoa1804980 30501490

[6] Giavridis T van der Stegen SJC Eyquem J Hamieh M Piersigilli A Sadelain M. CAR T Cell-Induced Cytokine Release Syndrome Is Mediated by Macrophages and Abated by IL-1 Blockade. Nat Med (2018) 24:731–8. 10.1038/s41591-018-0041-7 PMC6410714 29808005

[7] Brudno JN Kochenderfer JN. Recent Advances in CAR T-Cell Toxicity: Mechanisms, Manifestations and Management. Nat Med (2019) 34:45–55. 10.1016/j.blre.2018.11.002 PMC6628697 30528964

[8] Hay KA Hanafi LA Li D Gust J Liles WC Wurfel MM. Kinetics and Biomarkers of Severe Cytokine Release Syndrome After CD19 Chimeric Antigen Receptor-Modified T-Cell Therapy. Blood (2017) 130:2295–306. 10.1182/blood-2017-06-793141 PMC5701525 28924019

[9] Vercellino L Di Blasi R Kanoun S Tessoulin B Rossi C D’Aveni-Piney M. Predictive Factors of Early Progression After CAR T-Cell Therapy in Relapsed/Refractory Diffuse Large B-Cell Lymphoma. Blood Adv (2020) 4:5607–15. 10.1182/bloodadvances.2020003001 PMC7686887 33180899

[10] Qu C Ping N Kang L Liu H Qin S Wu Q. Radiation Priming Chimeric Antigen Receptor T-Cell Therapy in Relapsed/Refractory Diffuse Large B-Cell Lymphoma With High Tumor Burden. J Immunother (2020) 43:32–7. 10.1097/CJI.0000000000000284 31219975

[11] Sim AJ Jain MD Figura NB Chavez JC Shah BD Khimani F. Radiation Therapy as a Bridging Strategy for CAR T Cell Therapy With Axicabtagene Ciloleucel in Diffuse Large B-Cell Lymphoma. Int J Radiat Oncol Biol Phys (2019) 105:1012–21. 10.1016/j.ijrobp.2019.05.065 PMC6872916 31175906

[12] Wang J Mou N Yang Z Li Q Jiang Y Meng J. Efficacy and Safety of Humanized Anti-CD19-CAR-T Therapy Following Intensive Lymphodepleting Chemotherapy for Refractory/Relapsed B Acute Lymphoblastic Leukaemia. Br J Haematol (2020) 191:212–22. 10.1111/bjh.16623 PMC7687133 32232846

[13] Van Den Neste E Schmitz N Mounier N Gill D Linch D Trneny M. Outcomes of Diffuse Large B-Cell Lymphoma Patients Relapsing After Autologous Stem Cell Transplantation: An Analysis of Patients Included in the CORAL Study. Bone Marrow Transplant (2017) 52:216–21. 10.1038/bmt.2016.213 27643872

[14] Gutierrez M Chabner BA Pearson D Steinberg SM Jaffe ES Cheson BD. Role of a Doxorubicin-Containing Regimen in Relapsed and Resistant Lymphomas: An 8-Year Follow-Up Study of EPOCH. J Clin Oncol (2000) 18:3633–42. 10.1200/JCO.2000.18.21.3633 11054436

[15] Jermann M Jost LM Taverna C Jacky E Honegger HP Betticher DC. Rituximab-EPOCH, an Effective Salvage Therapy for Relapsed, Refractory or Transformed B-Cell Lymphomas: Results of a Phase II Study. Ann Oncol (2004) 15:511–6. 10.1093/annonc/mdh093 14998858

[16] Zelenetz AD Hamlin P Kewalramani T Yahalom J Nimer S Moskowitz CH. Ifosfamide, Carboplatin, Etoposide (ICE)-Based Second-Line Chemotherapy for the Management of Relapsed and Refractory Aggressive Non-Hodgkin’s Lymphoma. Ann Oncol (2003) 14 Suppl 1:i5–10. 10.1093/annonc/mdg702 12736224

[17] Vose J Sneller V. Outpatient Regimen Rituximab Plus Ifosfamide, Carboplatin and Etoposide (R-ICE) for Relapsed Non-Hodgkin’s Lymphoma. Ann Oncol (2003) 14 Suppl 1:i17–20. 10.1093/annonc/mdg704 12736226

[18] Kewalramani T Zelenetz AD Nimer SD Portlock C Straus D Noy A. Rituximab and ICE as Second-Line Therapy Before Autologous Stem Cell Transplantation for Relapsed or Primary Refractory Diffuse Large B-Cell Lymphoma. Blood (2004) 103:3684–8. 10.1182/blood-2003-11-3911 14739217

[19] Lopez A Gutierrez A Palacios A Blancas I Navarrete M Morey M. GEMOX-R Regimen Is a Highly Effective Salvage Regimen in Patients With Refractory/Relapsing Diffuse Large-Cell Lymphoma: A Phase II Study. Eur J Haematol (2008) 80:127–32. 10.1111/j.1600-0609.2007.00996.x 18005385

[20] El Gnaoui T Dupuis J Belhadj K Jais JP Rahmouni A Copie-Bergman C. Rituximab, Gemcitabine and Oxaliplatin: An Effective Salvage Regimen for Patients With Relapsed or Refractory B-Cell Lymphoma Not Candidates for High-Dose Therapy. Ann Oncol (2007) 18:1363–8. 10.1093/annonc/mdm133 17496309

[21] Corazzelli G Capobianco G Arcamone M Ballerini PF Iannitto E Russo F. Long-Term Results of Gemcitabine Plus Oxaliplatin With and Without Rituximab as Salvage Treatment for Transplant-Ineligible Patients With Refractory/Relapsing B-Cell Lymphoma. Cancer Chemother Pharmacol (2009) 64:907–16. 10.1007/s00280-009-0941-9 19219604

[22] Mounier N El Gnaoui T Tilly H Canioni D Sebban C Casasnovas RO. Rituximab Plus Gemcitabine and Oxaliplatin in Patients With Refractory/Relapsed Diffuse Large B-Cell Lymphoma Who Are Not Candidates for High-Dose Therapy. A Phase II Lymphoma Study Association Trial. Haematologica (2013) 98:1726–31. 10.3324/haematol.2013.090597 PMC3815173 23753028

[23] Liu P Liu M Lyu C Lu W Cui R Wang J. Acute Graft-Versus-Host Disease After Humanized Anti-CD19-CAR T Therapy in Relapsed B-ALL Patients After Allogeneic Hematopoietic Stem Cell Transplant. Front Oncol (2020) 10:573822. 10.3389/fonc.2020.573822 33117709 PMC7551306

[24] Cheson BD Fisher RI Barrington SF Cavalli F Schwartz LH Zucca E. Recommendations for Initial Evaluation, Staging, and Response Assessment of Hodgkin and non-Hodgkin Lymphoma: The Lugano Classification. J Clin Oncol (2014) 32:3059–68. 10.1200/JCO.2013.54.8800 PMC4979083 25113753

[25] Lee DW Gardner R Porter DL Louis CU Ahmed N Jensen M. Current Concepts in the Diagnosis and Management of Cytokine Release Syndrome. Blood (2014) 124:188–95. 10.1182/blood-2014-05-552729 PMC4093680 24876563

[26] Lee DW Santomasso BD Locke FL Ghobadi A Turtle CJ Brudno JN. ASTCT Consensus Grading for Cytokine Release Syndrome and Neurologic Toxicity Associated With Immune Effector Cells. Biol Blood Marrow Transplant. (2019) 25:625–38. 10.1016/j.bbmt.2018.12.758 PMC12180426 30592986

[27] Sesques P Ferrant E Safar V Wallet F Tordo J Dhomps A. Commercial Anti-CD19 CAR T Cell Therapy for Patients With Relapsed/Refractory Aggressive B Cell Lymphoma in a European Center. Am J Hematol (2020) 95:1324–33. 10.1002/ajh.25951 32744738

[28] Nastoupil LJ Jain MD Feng L Spiegel JY Ghobadi A Lin Y. Standard-Of-Care Axicabtagene Ciloleucel for Relapsed or Refractory Large B-Cell Lymphoma: Results From the US Lymphoma CAR T Consortium. J Clin Oncol (2020) 38:3119–28. 10.1200/JCO.19.02104 PMC7499611 32401634

[29] Locke FL Rossi JM Neelapu SS Jacobson CA Miklos DB Ghobadi A. Tumor Burden, Inflammation, and Product Attributes Determine Outcomes of Axicabtagene Ciloleucel in Large B-Cell Lymphoma. Blood Adv (2020) 4:4898–911. 10.1182/bloodadvances.2020002394 PMC7556133 33035333

[30] Ali SA Shi V Maric I Wang M Stroncek DF Rose JJ. T Cells Expressing an Anti-B-Cell Maturation Antigen Chimeric Antigen Receptor Cause Remissions of Multiple Myeloma. Blood (2016) 128:1688–700. 10.1182/blood-2016-04-711903 PMC5043125 27412889

[31] Brudno JN Maric I Hartman SD Rose JJ Wang M Lam N. T Cells Genetically Modified to Express an Anti-B-Cell Maturation Antigen Chimeric Antigen Receptor Cause Remissions of Poor-Prognosis Relapsed Multiple Myeloma. J Clin Oncol (2018) 36:2267–80. 10.1200/JCO.2018.77.8084 PMC6067798 29812997

