# Supplementary material for: Intensive Debulking Chemotherapy Improves the Short-Term and Long-Term Efficacy of Anti-CD19-CAR-T in Refractory/Relapsed DLBCL With High Tumor Bulk
Source: Front Oncol. 2021 Jul 30;11:706087. doi: 10.3389/fonc.2021.706087 (PMC8361834; doi:10.3389/fonc.2021.706087)
Supplement: Supplementary file 1 [file DataSheet_1.docx]

**Supplemental Table 1. Comparison of baseline characteristics between the chemotherapy and combined groups**

| Characteristics | The chemotherapy group | The combined group | *P value* |
| --- | --- | --- | --- |
| ***Median age (range)—year*** | 52(16-70) | 51(15-77) | 0.6402 |
| ***Male—no. (%)*** | 12(80%) | 18(72%) | 0.715 |
| ***Cell origin of cancer—no.(%)***  GCB  Non-GCB | 8(53%)  7(47%) | 11(44%)  14(56%) | 0.745 |
| ***Disease stage at study entry—no. (%)***  III  IV | 7(47%)  8(53%) | 7(28%)  18(72%) | 0.310 |
| ***IPI scores at study entry—no. (%)***  2 points  3 points  4 points  5 points | 1(7%)  11(73%)  2(13%)  1(7%) | 4(16%)  16(64%)  4(16%)  1(4%) | 0.680 |
| ***No. of previous lines of antineoplastic therapy—no. (%)***  Medium(range) | 3(2-4) | 3(2-4) | 0.9185 |
| ***Medium tumor diameter (range)—cm*** | 8.3(7.6-13.6) | 8.8(7.5-20.0) | 0.0735 |

**Supplemental Table 2. Debulking chemotherapy in patients of the combined group**

| Patient number | Chemotherapy regimen | Tumor bulk shrunk or not | Potassium  (mmol/L) | Calcium  (mmol/L) | Phosphorus  (mmol/L) | Uric acid  (μmol/L) | Urea nitrogen  (mmol/L) | Creatinine  (μmol/L) |
| --- | --- | --- | --- | --- | --- | --- | --- | --- |
| P1^#^ | DHAP | Y | 4.94 | 2.01 | 1.10 | 148.00 | 1.97 | 57.00 |
| P2^#^ | DHAP | N | 5.04 | 1.90 | 1.30 | 336.10 | 8.12 | 104.00 |
| P3^#^ | ICE | Y | 3.71 | 2.17 | 1.59 | 331.30 | 4.46 | 60.00 |
| P4^#^ | DHAP | Y | 3.85 | 2.12 | 1.22 | 305.60 | 4.25 | 68.00 |
| P5^#^ | DHAP | Y | 3.77 | 2.08 | 0.96 | 409.20 | 5.56 | 79.00 |
| P6^#^ | DHAP | N | 3.85 | 2.05 | 1.05 | 319.40 | 1.55 | 50.00 |
| P7^#^ | DA-EPOCH | Y | 4.24 | 2.22 | 1.01 | 218.10 | 5.19 | 77.00 |
| P8^#^ | DHAP | Y | 4.20 | 2.21 | 0.92 | 315.00 | 3.16 | 54.00 |
| P9^#^ | ICE | Y | 4.04 | 1.91 | 0.94 | 264.80 | 5.37 | 61.00 |
| P10^#^ | DHAP | Y | 4.49 | 2.42 | 1.06 | 156.70 | 9.73 | 65.00 |
| P11^#^ | DHAP | Y | 3.47 | 1.98 | 1.09 | 241.20 | 6.47 | 70.00 |
| P12^#^ | DHAP | Y | 5.35 | 2.30 | 1.47 | 405.30 | 3.21 | 73.00 |
| P13^#^ | ICE | Y | 3.72 | 2.27 | 1.30 | 250.60 | 2.41 | 47.00 |
| P14^#^ | DHAP | N | 3.97 | 2.03 | 1.72 | 302.90 | 8.26 | 85.00 |
| P15^#^ | DHAP | Y | 4.47 | 2.07 | 1.13 | 186.30 | 4.43 | 64.00 |
| P16^#^ | DHAP | N | 3.64 | 2.25 | 1.24 | 106.50 | 3.14 | 43.00 |
| P17^#^ | DHAP | Y | 4.37 | 2.04 | 1.36 | 105.60 | 1.54 | 35.00 |
| P18^#^ | ICE | N | 4.18 | 2.19 | 1.17 | 197.10 | 7.31 | 70.00 |
| P19^#^ | DHAP | Y | 4.29 | 1.95 | 2.64 | 801.90 | 23.53 | 129.00 |
| P20^#^ | ICE | Y | 4.12 | 2.12 | 1.16 | 273.40 | 3.69 | 49.00 |
| P21^#^ | ICE | Y | 4.73 | 2.06 | 1.46 | 230.90 | 2.05 | 50.00 |
| P22^#^ | ICE | Y | 3.84 | 2.01 | 1.26 | 423.10 | 4.67 | 58.00 |
| P23^#^ | DHAP | N | 3.97 | 2.21 | 1.30 | 179.70 | 5.66 | 44.00 |
| P24^#^ | ICE | Y | 3.90 | 2.24 | 1.38 | 228.40 | 4.56 | 51.00 |
| P25^#^ | GemOx | Y | 4.84 | 2.39 | 1.48 | 288.20 | 4.40 | 55.00 |
|  |  | **mean ± *SD*** | 4.20±0.48 | 2.13±0.14 | 1.29±0.35 | 281.01±139.26 | 5.39±4.34 | 63.92±20.29 |

**Supplemental Table 3. Comparison of baseline characteristics between the chemo-sensitive and chemo-refractory groups**

| Characteristics | The chemo-sensitive group | The chemo-refractory group | *P value* |
| --- | --- | --- | --- |
| ***Median age (range)—year*** | 51(15-77) | 51(39-68) | 0.9941 |
| ***Male—no. (%)*** | 15(79%) | 3(50%) | 0.298 |
| ***Cell origin of cancer—no.(%)***  GCB  Non-GCB | 9(47%)  10(53%) | 2(33%)  4(67%) | 0.661 |
| ***Disease stage at study entry—no. (%)***  III  IV | 7(37%)  12(63%) | 0(0%)  6(100%) | 0.137 |
| ***IPI scores at study entry—no. (%)***  2 points  3 points  4 points  5 points | 4(21%)  11(58%)  3(16%)  1(5%) | 0(0%)  5(83%)  1(17%)  0(0%) | 0.687 |
| ***No. of previous lines of antineoplastic therapy—no. (%)***  Medium(range) | 3(2-4) | 2.5(2-4) | 0.6618 |
| ***Medium tumor diameter (range)—cm*** | 8.6(7.5-20.0) | 10.4(7.8-13.4) | 0.7298 |

**Supplemental Table 4. Minimum values of neutrophil, hemoglobin and platelet after debulking chemotherapy, on the day of CAR-T infusion and after CAR-T infusion in the combined group**

| Patient number | Minimum value of neutrophil, hemoglobin and platelet after debulking chemotherapy | | | Value of neutrophil, hemoglobin and platelet on the day of CAR-T infusion | | | Minimum value of neutrophil, hemoglobin and platelet after CAR-T infusion | | |
| --- | --- | --- | --- | --- | --- | --- | --- | --- | --- |
|  | neutrophil | hemoglobin | platelet | neutrophil | hemoglobin | platelet | neutrophil | hemoglobin | platelet |
| P1# | **1.46** | **110** | **156** | **1.46** | **110** | **156** | **0.14** | **75** | **23** |
| P2^#^ | 2.38 | 100 | 143 | 2.38 | 100 | 143 | 0.25 | 83 | 24 |
| P3^#^ | 1.13 | 119 | 42 | 1.13 | 119 | 42 | 0.36 | 113 | 27 |
| P4^#^ | 2.23 | 85 | 212 | 2.31 | 85 | 258 | 0.85 | 88 | 227 |
| P5^#^ | 1.48 | 118 | 59 | 1.48 | 126 | 59 | 0.50 | 116 | 50 |
| P6^#^ | 1.79 | 93 | 222 | 1.79 | 93 | 222 | 0.61 | 84 | 146 |
| P7^#^ | 0.18 | 95 | 118 | 0.18 | 95 | 118 | 1.11 | 84 | 127 |
| P8^#^ | 1.36 | 117 | 234 | 1.48 | 117 | 234 | 1.36 | 111 | 263 |
| P9^#^ | 3.82 | 83 | 172 | 5.63 | 92 | 207 | 0.43 | 66 | 88 |
| P10^#^ | 0.57 | 68 | 13 | 0.57 | 72 | 13 | 0.03 | 59 | 3 |
| P11^#^ | 2.49 | 96 | 220 | 2.49 | 97 | 220 | 0.03 | 82 | 36 |
| P12^#^ | 4.10 | 105 | 670 | 4.10 | 105 | 670 | 0.13 | 85 | 539 |
| P13^#^ | 1.52 | 116 | 104 | 1.65 | 116 | 104 | 0.10 | 111 | 70 |
| P14^#^ | 1.36 | 110 | 56 | 1.36 | 110 | 56 | 0.48 | 78 | 2 |
| P15^#^ | 1.56 | 125 | 80 | 1.56 | 130 | 80 | 0.02 | 97 | 6 |
| P16^#^ | 0.60 | 92 | 204 | 0.74 | 110 | 204 | 1.16 | 104 | 167 |
| P17^#^ | 1.06 | 92 | 160 | 1.06 | 92 | 160 | 0.12 | 90 | 143 |
| P18^#^ | 1.48 | 98 | 145 | 1.48 | 106 | 145 | 0.77 | 91 | 69 |
| P19^#^ | 1.46 | 109 | 32 | 2.94 | 115 | 32 | 0.03 | 87 | 17 |
| P20^#^ | 1.11 | 83 | 152 | 1.29 | 87 | 152 | 0.76 | 89 | 102 |
| P21^#^ | 0.33 | 74 | 29 | 0.55 | 74 | 29 | 0.31 | 67 | 29 |
| P22^#^ | 0.23 | 81 | 64 | 0.23 | 81 | 64 | 0.01 | 60 | 14 |
| P23^#^ | 1.57 | 75 | 246 | 1.57 | 76 | 246 | 0.17 | 70 | 49 |
| P24^#^ | 1.25 | 86 | 228 | 1.83 | 87 | 228 | 0.89 | 79 | 60 |
| P25^#^ | 3.62 | 119 | 364 | 3.62 | 119 | 364 | 3.46 | 124 | 159 |
